# Comprehensive Bioinformatic Analysis and Expression Profiling of the Alcohol Dehydrogenase (*ADH*) Gene Family in *Morus alba*

**DOI:** 10.3390/biology15201808

**Published:** 2026-10-10

**Authors:** Ju Tang, Mingyue Xie, Minxiao Zhou, Shenghao Zhang, Yanjie Li, Jingtao Hu

**Affiliations:** College of Biology and Food Engineering, Chongqing Sanxia University of Science and Technology, Chongqing 404100, China

**Keywords:** genome-wide analysis, alcohol dehydrogenase (*ADH*), abiotic stress, gene expression, *Morus alba*

## Abstract

Alcohol dehydrogenase (*ADH*) is a key enzyme for ethanol fermentation under anaerobiosis and is vital for plant growth, development, and abiotic stress tolerance. Although the (*MDR*)-*ADH* gene family in *Morus* has not been systematically characterized, this study performs a genome-wide analysis to investigate its evolution and flooding-responsive transcription patterns. In this study, a comprehensive genome-wide bioinformatic analysis of the *Morus alba* (*MDR*)-*ADH* (*MaADH*) gene family, including physicochemical characteristics, evolutionary relationships, subcellular localization, gene structural organization, cis-regulatory element composition, and interspecific syntenic relationships, was conducted. A total of 20 *MaADH* genes were identified from the *M. alba* genome, and all encoded proteins contained the conserved GroES-like alcohol dehydrogenase N-terminal domain (ADH_N) and zinc-binding alcohol dehydrogenase domain (ADH_zinc_N). Phylogenetic analysis grouped these genes into six evolutionary clades. The identified *MaADH* genes showed an uneven chromosomal distribution across seven chromosomes. Moreover, qRT-PCR analysis of 11 selected *MaADH* genes showed that most genes are significantly upregulated in the roots and stems under flooding conditions, with a characteristic temporal expression pattern of initial induction followed by attenuation. These results suggest that the differentially expressed *MaADH* genes may participate in the transcriptional response to flooding stress in *M. alba*. These results will contribute to further research on the biological function of the *MaADH* candidates and provide new candidate genes for breeding programs focused on the flood response of *M. alba*.

## Simple Summary

Alcohol dehydrogenase (*ADH*) genes are critical for plant stress adaptation, particularly under flooding. Here, we identified 20 (*MDR*)-*ADH* family genes in *Morus alba* and analyzed their physicochemical characteristics, phylogeny, gene structure, cis-acting elements, and interspecific synteny. Transcriptome analysis combined with qRT-PCR validation was performed to explore the expression profiles of *MaADH* genes under flooding stress. *MaADH* members exhibited distinct tissue- and time-specific expression patterns, indicating their potential roles in mulberry flooding tolerance. This study provides valuable gene resources and a theoretical foundation for further functional investigation and flooding-resistance breeding of mulberry.

## 1. Introduction

Alcohol dehydrogenase (ADH; EC 1.1.1.1) is a zinc-dependent oxidoreductase in the extensively characterized medium-chain dehydrogenase/reductase (MDR) superfamily [1]. The enzyme comprises two functional domains: a catalytic/substrate-binding domain that contains the active-site zinc ion and a coenzyme-binding domain with a Rossmann fold that specifically interacts with NAD^+^/NADH cofactors [2]. ADH catalyzes the reversible oxidation–reduction reaction between ethanol and acetaldehyde, as well as related alcohol/aldehyde substrates, via NAD^+^/NADH-dependent redox conversion. This reaction represents the terminal step of ethanolic fermentation during glycolytic metabolism [3]. ADH proteins are widely distributed in different organisms and have been classified into three major subfamilies based on polypeptide chain length: short-chain dehydrogenase/reductases (SDR-ADHs), which generally contain approximately 250 amino acid residues; medium-chain dehydrogenase/reductases (MDR-ADHs), which typically consist of 350 amino acid residues; and long-chain dehydrogenase/reductases (LDR-ADHs), which possess protein lengths ranging from 600 to 750 amino acid residues [4,5,6]. Previous studies have documented that the catalytic regions of ADH proteins contain conserved zinc-binding residues essential for enzymatic activity [7,8,9]. As a major component of the MDR protein superfamily, ADHs include diverse members, such as animal and plant ADHs, as well as glutathione-dependent formaldehyde dehydrogenase (FADH), which is also recognized as a class III ADH [6]. Studies have shown that most members of the *ADH* gene family identified in plants belong to the (MDR)-ADH subfamily [7,8,9].

The *ADH* gene family plays a crucial role in regulating plant growth, development, and environmental adaptation. When plants encounter conditions that limit oxygen availability, such as flooding, cellular respiration shifts from aerobic metabolism to anaerobic pathways, leading to increased ethanol production. *ADH* is a key enzyme in the ethanolic fermentation pathway and is involved in metabolic reprogramming under oxygen-deficient conditions by maintaining energy production and cellular homeostasis [10]. In *Arabidopsis thaliana*, *Glycine max*, and *Panax ginseng*, the *ADH* gene is involved in responses to abiotic stresses such as hypoxia, flooding, and ABA, leading to different, tissue-specific expression patterns in plants [11,12,13]. Under flooding stress, ADH catalyzes the reduction of acetaldehyde to ethanol while simultaneously oxidizing NADH to NAD^+^, ensuring that glycolysis continues under anaerobic conditions and providing the plant with substrate-level ATP [14]. In the flood-tolerant tree species *Taxodium distichum*, *TdADHA3* was significantly upregulated after 24 h of flooding and is considered a key gene for ethanol fermentation and flood tolerance [15]. In *Zanthoxylum armatum*, overexpression of *ZaADH1* enhanced tolerance to flooding stress by increasing soluble sugar and trehalose levels and promoting glycolysis and carbohydrate metabolism [16]. In *Triticum aestivum*, members of the *TaADH* gene family show differential transcriptional responses between flooding-tolerant and flooding-sensitive cultivars after flooding, indicating their potential role in stress adaptation [17]. Similarly, in *Cucumis melo*, individual *CmADH* genes show different expression profiles in response to phytohormonal treatments and show tissue-dependent regulation, suggesting functional diversification among family members [18].

*Morus alba* L. is a perennial woody species in the genus *Morus* of the family Moraceae. This economically important plant has diverse tissues with significant agricultural and industrial applications (such as silkworm rearing using mulberry leaves and the textile industry). However, Mulberry orchards in rainy regions or low-lying areas often face the risk of short-term seasonal flooding, which can inhibit root activity and reduce leaf area, ultimately leading to a decline in leaf yield and quality [19,20,21]. Although the *ADH* gene has been widely reported in the flooding responses of various plants, no studies have yet been published that analyze the (*MDR*)-*ADH* gene family in mulberry at the genomic level; therefore, a systematic analysis of the medium-chain *ADH* gene family in mulberry is of great importance. In this study, we aim to perform a genome-wide identification of the (*MDR*)-*ADH* gene family in *M. alba* and characterize their gene structures, phylogenetic features, conserved motifs, cis-regulatory elements, and interspecies synteny. We also seek to examine the expression profiles of *MaADH* genes under flooding stress. This study will deepen our understanding of the evolution of the *MaADH* family and may provide candidate genes for further research into the mechanisms by which *M. alba* adapts to flooding.

## 2. Materials and Methods

### 2.1. Plant Cultivation and Abiotic Stress Treatment

Seedlings of the mulberry cultivar ‘Yu 711’ (*Morus alba*), approximately 30 cm in height with stem diameters of 0.2–0.4 cm, were individually grown in pots (21 cm in diameter × 21 cm in height; one seedling per pot) filled with a substrate mixture of nutrient soil, vermiculite, and loess. The plants were maintained in a greenhouse at 25 °C, 16 h light/8 h dark photoperiod, and 75% relative humidity for 45 days before the stress treatment.

Place plants with similar growth conditions in a water-filled tank, submerging the pots so that the water level is about 10 cm above the soil surface. Place one pot per tank to subject the plants to flooding stress. Seedlings grown under normal conditions without flooding served as the 0 d control group. At each sampling time point (0, 1, 3, and 7 d after flooding) [22], each tank represented one independent biological replicate; three independent biological replicates were prepared for each tissue type (root, stem, and leaf). Each biological replicate consisted of pooled tissues collected from three individual seedlings, with one seedling grown per pot. Root, stem, and leaf tissues were harvested separately at each time point, immediately frozen in liquid nitrogen, and stored at −80 °C until RNA extraction and qPCR analysis.

### 2.2. Identification of the ADH Gene Family in M. alba

Genome sequence data for *Morus alba* (accession number GCA_012066045.3) were obtained from the National Center for Biotechnology Information (NCBI). The genome assembly of *M. alba* (GCA_012066045.3) was annotated using Helixer (v0.3.4), with the “land_ plant” model and default parameter settings (https://www.plabipd.de/helixer_main.html, accessed on 30 March 2025) [23]. Hidden Markov Model (HMM) profiles for the conserved ADH domains PF00107 (ADH_zinc_N) and PF08240 (ADH_N) were retrieved from the Pfam database (http://www.ebi.ac.uk/interpro/entry/pfam/#table, accessed on 26 November 2025). Candidate ADH family members were identified by performing BLAST alignment searches for Arabidopsis and rice in TBtools-II (v2.485) with an E-value of 1 × 10^−5^ [24]. The resulting candidate protein sequences were further validated for conserved domains using the Conserved Domain Database (CDD) (https://www.ncbi.nlm.nih.gov/Structure/bwrpsb/bwrpsb.cgi, accessed on 27 November 2025) [25] and the SMART database (https://smart.embl.de/, accessed on 27 November 2025) [26]. The ADH_N domain represents the conserved catalytic region of *ADH*, whereas the ADH_zinc_N domain corresponds to the zinc-binding region required for catalytic activity. Only proteins containing both conserved domains were considered authentic members of the (*MDR*)-*ADH* gene family. Subsequently, redundant sequences, proteins with incomplete domain architecture, and sequences with abnormal protein lengths were manually removed. Finally, the retained genes were designated as *MaADH* genes. The physicochemical properties of MaADH proteins, including amino acid length, grand average of hydropathicity (GRAVY), theoretical isoelectric point (pI), and molecular weight (MW), were calculated using TBtools-II (v2.485) [24]. Subcellular localization was predicted using WoLF PSORT (https://wolfpsort.hgc.jp/, accessed on 2 December 2025), and protein secondary structure was analyzed using SOPMA (https://npsa.lyon.inserm.fr/cgi-bin/npsa_automat.pl?page=/NPSA/npsa_sopma.html, accessed on 2 December 2025).

### 2.3. Multiple Sequence Alignment and Evolutionary Relationship Analysis of the MaADH Gene Family

ADH protein sequences from grapevine (*Vitis vinifera*) were retrieved from the NCBI database using gene identifiers reported in previous wheat-related studies [17]. Other ADH protein sequences from *A. thaliana* [13], *Solanum lycopersicum* [27], *T. aestivum* [17], and *C. melo* [18] were also obtained from the NCBI database. Multiple sequence alignment of MaADH protein sequences was performed using Clustal W in MEGA 7.0 (v7.0.26). Poorly aligned regions were manually trimmed. A maximum-likelihood phylogenetic tree was constructed in MEGA 7.0 using the LG + G4 amino acid substitution model, with pairwise deletion for gap handling and 1000 bootstrap replicates. The tree was unrooted and visualized with midpoint rooting for display. The tree was visualized and decorated using Evolview (https://www.evolgenius.info/evolview/, accessed on 19 September 2026).

### 2.4. Chromosomal Localization Analysis of the MaADH Genes

To examine the chromosomal distribution patterns of the identified *MaADH* genes, chromosomal positions were obtained from the *M. alba* genome annotation files. A chromosome localization map showing the physical positions of *MaADH* genes was generated using TBtools-II (v2.485) [24].

### 2.5. Conserved Motif and Gene Structure Characterization of the MaADH Gene Family

The exon–intron organization of *MaADH* genes was determined from the *M. alba* genome annotation and visualized using TBtools-II (v2.485) [24]. Conserved motif analysis of MaADH protein sequences was performed using the MEME Suite online platform (https://meme-suite.org/meme/tools/meme, accessed on 16 December 2025), with the maximum number of motifs set to 10. The identified conserved motifs were analyzed and visualized with TBtools-II (v2.485) to generate motif distribution patterns for the MaADH protein family [24].

### 2.6. Promoter Cis-Regulatory Element Analysis of the MaADH Genes

To explore the potential transcriptional regulatory characteristics of *MaADH* genes, cis-element prediction was conducted. The 2000 bp genomic sequence upstream of the translation initiation site (ATG) of each *MaADH* gene was extracted and defined as the candidate promoter region due to the lack of accurate transcription start site (TSS) annotation in the *M. alba* genome. The predicted promoter sequences were analyzed using the PlantCARE database (https://ngdc.cncb.ac.cn/databasecommons/database/id/5261, accessed on 6 January 2026) to identify potential cis-acting regulatory elements [28] and visualized using TBtools-II (v2.485) [24].

### 2.7. Collinearity Analysis of the MaADH Gene

To examine the evolutionary conservation and genomic relationships of the *MaADH* gene, comparative synteny analysis was conducted between *M. alba* and six representative plant species. Genome sequences and corresponding annotation files of three dicotyledonous species, including *A. thaliana*, *Populus trichocarpa*, and *C. melo*, as well as three monocotyledonous species, including *Zea mays*, *Oryza sativa*, and *T. aestivum*, were downloaded from the Ensembl Plants FTP database. The syntenic relationships between homologous *ADH* genes in *M. alba* and the selected plant species were analyzed using the OneStepMCScanX model implemented in TBtools-II (v2.485) [24].

### 2.8. Transcriptome Sequencing Analysis of MaADH Gene Expression Patterns Under Different Stress Treatments

To investigate the expression patterns of the *MaADH* gene in mulberry seedlings under various stress treatments (ABA, MeJA, drought, salinity, and flooding), we extracted the FPKM values for the *MaADH* gene from root and leaf tissues of mulberry seedlings at different time points under various stress conditions using RNA-Seq data (SRP737661) (Table S1).

### 2.9. qRT-PCR Expression Analysis

Total RNA was extracted from collected plant tissues using TRIzol reagent (Takara, Dalian, China) according to the manufacturer’s protocol. RNA integrity assessment and cDNA synthesis were performed according to the method described by Hu et al. (2023) [22]. Gene-specific primers for qRT-PCR analysis of selected *MaADH* genes were designed using Primer3 software (Primer3, Vancouver, WA, Canada). The primer sequences used in this study are listed in Supplementary Table S2. Using *MaActin* as the internal control, RT-qPCR experiments were performed on a fully automated medical PCR analysis system using the Azaood Universal SYBR Green qPCR Mix (Azaood, Birmingham, AL, USA). The total reaction volume was 20 μL, containing a standardized cDNA template, specific primers, and PCR buffer. The amplification program was set as follows: 3 min of pre-denaturation at 95 °C, followed by 40 cycles, each consisting of 10 s at 95 °C and 30 s at 60 °C. Melting curve analysis was performed within the range of 65 °C to 95 °C to verify the specificity of the amplification products. The relative expression level of the *MaADH* gene was determined using the 2^−ΔΔCt^ method based on raw Cq values [29]. Three technical replicates were performed for each sample.

### 2.10. Statistical Analysis

Two-way analysis of variance (ANOVA) followed by Dunnett’s multiple-comparison test was performed using GraphPad Prism 10.0 (GraphPad Software, Boston, MA, USA). Statistical analyses were conducted on ΔCq values rather than fold-change values. Within each tissue type, each flooding time point was compared against the 0-day control. Differences were considered statistically significant at *p* < 0.05. Data are presented as mean ± standard deviation (mean ± SD), and bar charts were generated accordingly.

## 3. Results

### 3.1. Identification and Characterization of the MaADH Gene

After rigorous domain-based screening to confirm the presence of both conserved ADH domains (Figure 1) and remove incomplete or potentially non-functional sequences, a total of 20 *MaADH* genes were identified from the *M. alba* genome. These genes were designated as *MaADH1* to *MaADH20* according to their physical chromosomal positions (Table S3). The encoded MaADH proteins showed considerable variation in their physicochemical properties. Protein length ranged from 306 amino acids (MaADH15) to 635 amino acids (MaADH10), corresponding to predicted molecular weights of 32.71 kDa (MaADH15) to 68.29 kDa (MaADH10). The theoretical isoelectric point (pI) ranged from 5.28 to 9.05, with an average of 6.75. Among the identified proteins, 15 members were acidic (pI < 7), whereas five were classified as basic proteins. Hydropathicity analysis revealed negative GRAVY values for six MaADH proteins, indicating relatively hydrophilic characteristics. Subcellular localization predictions suggested that most MaADH proteins were localized to the cytoplasm. However, MaADH8 and MaADH13 were predicted to localize to chloroplasts, whereas MaADH10 was predicted to target plastids. Secondary structure prediction indicated that MaADH proteins primarily consisted of α-helices, β-sheets, and random-coil regions, suggesting conserved structural characteristics among family members.

### 3.2. Phylogenetic Relationships and Homology Analysis of the MaADH Gene

To investigate evolutionary relationships and potential functional divergence among members of the *MaADH* gene family in *M. alba*, a phylogenetic analysis was conducted using the 20 identified MaADH proteins and ADH from five representative plant species, including *A. thaliana* (7 members), *S. lycopersicum* (7 members), *T. aestivum* (22 members), *C. melo* (13 members), and *V. vinifera* (8 members). A total of 77 ADH protein sequences were included in the phylogenetic analysis (Figure 2; Table S4). Based on amino acid sequence homology, all ADH proteins are classified into six subfamilies (I–VI). Among these 20 mulberry MaADH proteins, MaADH10, MaADH8, and MaADH16 belong to subfamilies I, II, and V, respectively; MaADH14 and MaADH15 belong to subfamily III; and the remaining 15 proteins all belong to subfamily IV. There are no MaADHs in subfamily VI. All MaADHs are medium-chain ADH proteins.

A group of MaADH proteins, including MaADH6, MaADH7, MaADH11, MaADH18, MaADH19, and MaADH20, clustered within a single phylogenetic branch, indicating a close evolutionary relationship among these members. To further evaluate the conservation of amino acid sequences within this subgroup, a multiple sequence alignment was performed using *A. thaliana* ADH1 (At-ADH1) as the reference sequence (Figure 3).

The sequence alignment analysis showed an overall amino acid identity of 39.46% between these six MaADH proteins and At-ADH1. All analyzed proteins retained the highly conserved Zn1 motif [GHE(X)_2_G(X)_5_G(X)_2_V], the Zn2 motif [GD(X)_9–10_C(X)_2_C(X)_2_C(X)_2_C(X)_7_C], and the putative dinucleotide-binding motif [GXG(X)_2_G]. The putative dinucleotide-binding motif corresponds to the Rossmann fold, which is characteristic of the ADH_N domain. A separate zinc-binding motif is responsible for metal coordination.

Similarly, the more distantly related MaADH proteins, including MaADH1, MaADH2, MaADH3, MaADH4, MaADH5, MaADH9, MaADH12, and MaADH17, were aligned with At-ADH1. These proteins showed an overall sequence identity of 40.28% and conserved the three characteristic functional motifs, including the zinc-binding regions and the putative dinucleotide-binding motif domain. The preservation of these motifs further supports their classification within the MDR-ADH subfamily and suggests the maintenance of essential catalytic functions throughout evolutionary diversification.

### 3.3. Chromosomal Localization Analysis of the MaADH Gene and Gene Duplication Events

The chromosomal distribution pattern of the 20 identified *MaADH* genes was analyzed and visualized using a chromosome localization map (Figure 4). The results showed that *MaADH* genes exhibited uneven, irregular distribution across seven chromosomes, including Chr5-Chr8 and Chr11-Chr13. Among the analyzed chromosomes, Chr8 and Chr12 each contained only one *MaADH* gene, corresponding to *MaADH12* and *MaADH17*, respectively. In comparison, Chr5 contained the highest number of *MaADH* genes, with five, followed by Chr11, which contained four. Chr6, Chr7, and Chr13 each contained three *MaADH* genes. Most *MaADH* genes were located near the terminal regions of their respective chromosomes.

A tandem duplication event consists of two or more chromosomal regions within 200 kb of each other in the same genomic region. Further analysis of gene duplication events identified four tandem duplication clusters involving eight *MaADH* genes distributed across three chromosomes (Chr5, Chr6, and Chr11). Two tandem duplication events were detected on Chr5, involving the gene pairs *MaADH1/MaADH2* and *MaADH4/MaADH5*, respectively. There is one pair each on Chr6 and Chr11: *MaADH6/MaADH7* and *MaADH14/MaADH15*, respectively.

### 3.4. Conserved Motif and Gene Structure Characterization of the MaADH Gene

To investigate structural conservation and functional diversification of the *MaADH* gene family, conserved motif analysis was conducted across the 20 identified MaADH proteins (Figure 5). Using the MEME online platform, 10 conserved motifs were identified and labeled motif1 to motif10 (Figure 5A; Table S5). The amino acid sequences and corresponding sequence logos for these motifs are presented in Figure S1. The identified conserved motifs ranged from 18 to 50 amino acids in length. Among these motifs, motif 3 was the shortest, whereas motifs 2, 4, 5, and 8 each contained 50 amino acid residues. Motif 1 and motif 2 contained the characteristic conserved signatures [GHE(X)_2_G(X)_5_G(X)_2_V] and [GXG(X)_2_G], respectively, corresponding to the zinc-binding catalytic region and the putative dinucleotide-binding motif domain of ADH proteins. All identified MaADH proteins contained motif 2, indicating universal conservation of the putative dinucleotide-binding motif region across the family. Motif 1 was absent only in MaADH8 and MaADH13, whereas motif 3 was absent only in MaADH13. These variations suggest structural diversification among specific MaADH members.

Analysis of motif arrangements revealed that 60% of the MaADH proteins exhibited a highly conserved motif organization pattern represented by “3–1–6–7–2–10–9–5”, whereas motifs 4 and 8 were absent in this subgroup. Although most members showed similar motif arrangements, several proteins exhibited distinct structural configurations. MaADH3 and MaADH8 showed significant differences in motif positioning compared with other family members. Six proteins, including MaADH6, MaADH7, MaADH11, MaADH18, MaADH19, and MaADH20, shared a unique motif arrangement pattern of “10–3–1–6–7–2–8–4”, with motifs 5 and 9 absent from these proteins. The consistent conservation of this motif architecture, together with their clustering within the same phylogenetic branch, indicates a close evolutionary relationship and suggests possible functional similarity among these six MaADH members.

To further characterize the structural diversity of the *MaADH* gene family, the exon–intron organization was analyzed using genomic annotation data (Figure 5B). All identified *MaADH* genes contained introns, confirming their conserved eukaryotic gene architecture. Most *MaADH* genes had two untranslated regions (UTRs), whereas *MaADH10* and *MaADH13* had only one UTR. The number of exons varied among *MaADH* members, ranging from four to 18 exons per gene. Two distinct structural patterns were identified within the family. Six genes, including *MaADH6*, *MaADH7*, *MaADH11*, *MaADH18*, *MaADH19*, and *MaADH20*, exhibited a conserved structural organization consisting of five exons and four introns. Another six members, including *MaADH1*-*MaADH5* and *MaADH17*, consistently showed a structure with 10 exons and nine introns.

### 3.5. Analysis of Cis-Regulatory Elements of the MaADH Gene

To elucidate the potential transcriptional regulatory mechanisms of *MaADH* genes, cis-acting element analysis was performed based on the 2000 bp upstream candidate promoter regions of *MaADH* genes (Figure 6; Figure S2; Table S6). Numerous stress- and hormone-responsive cis-elements were identified, implying the potential involvement of *MaADH* genes in environmental stress responses. Among these regulatory elements, light-responsive motifs were the most abundant category, comprising 23 elements (Figure S3). Box-4 and G-box elements were widely distributed across nearly all *MaADH* promoter regions, indicating their potential involvement in light-mediated transcriptional regulation. Several stress-associated cis-elements were also identified, including anaerobic-responsive elements (ARE), hypoxia-responsive elements, and defense/stress-related regulatory motifs. Among these, 43 ARE elements associated with anaerobic induction were detected, suggesting that *MaADH* genes may be transcriptionally regulated under oxygen-deficient conditions. Hormone-responsive regulatory elements were also widely distributed across *MaADH* promoters. Auxin-responsive elements, particularly TGA-element motifs, were detected in most *MaADH* members. Abscisic acid (ABA)-responsive ABRE elements were identified 45 times across the analyzed promoter regions and were present in most genes, although they were absent from *MaADH8*, *MaADH11*, *MaADH19*, and *MaADH20*. MeJA-responsive motifs, including CGTCA- and TGACG-motif elements, accounted for 46.10% of the identified hormone-responsive elements. Only 20% of *MaADH* genes lacked MeJA-associated regulatory motifs. Elements associated with plant growth, developmental regulation, and MYB transcription factor binding were detected in the promoter regions. The diverse composition and wide distribution of cis-regulatory elements within the candidate promoter regions of *MaADH* genes imply that members of this gene family may be involved in complex regulatory networks associated with developmental processes, hormonal signaling, and abiotic stress adaptation in *M. alba*.

### 3.6. Analysis of Synteny in the MaADH Gene

To observe the evolutionary conservation and diversification patterns of the *ADH* gene family among different angiosperm lineages, comparative synteny analysis was performed between *M. alba* and six representative plant species comprising three monocotyledonous species (*Z. mays*, *O. sativa*, and *T. aestivum*) and three dicotyledonous species (*A. thaliana*, *P. trichocarpa*, and *C. melo*) (Figure 7; Table S7). The comparative genomic analysis revealed distinct syntenic relationships between *MaADH* genes and their homologs in the selected plant species. A total of 1, 3, and 7 homologous *ADH* gene pairs were identified between *M. alba* and maize, rice, and wheat, respectively. Among the examined species, *M. alba* exhibited the most syntenic relationships with *P. trichocarpa*, with 13 conserved homologous gene pairs identified. This was followed by *A. thaliana* with 10 syntenic pairs and *C. melo* with 6 syntenic pairs. A higher number of conserved syntenic relationships observed between *M. alba* and dicotyledonous species than between *M. alba* and monocotyledonous species suggests a closer evolutionary relationship and a higher degree of genomic conservation between mulberry and other dicot lineages.

### 3.7. Tissue-Specific Expression Analysis of the MaADH Gene

To examine the potential biological functions of *MaADH* genes during mulberry growth and development, the expression patterns of 11 selected *MaADH* genes, including *MaADH1*, *MaADH3*, *MaADH4*, *MaADH5*, *MaADH6*, *MaADH7*, *MaADH11*, *MaADH13*, *MaADH14*, *MaADH17*, and *MaADH20*, were examined in roots, stems, and leaves (Figure 8). The expression profiles showed considerable tissue-specific variation among the *MaADH* genes analyzed. With the exception of *MaADH1*, the expression levels of the other 10 genes were higher in leaf tissue than in root and stem tissues.

### 3.8. Expression Analysis of MaADH Genes Under Different Stress Conditions

To further observe the roles of *MaADH* genes, the FPKM values derived from transcriptomic data (SRP737661) were used to analyze the transcriptional expression patterns of *MaADH* family members under ABA, MeJA, drought, flooding, and salt stress (Figure 9; Table S1). Under phytohormone treatments, none of the analyzed *MaADH* genes showed elevated relative expression compared with the control. Nevertheless, *MaADH7* and *MaADH18* exhibited reduced expression at 3 h after hormone application. Under abiotic stress conditions, *MaADH* genes displayed varied expression levels. After 1 day of flooding and salt treatments, the relative expression of *MaADH1* and *MaADH14* increased, while *MaADH3*, *MaADH4*, and *MaADH7* showed decreased expression. Under drought stress, several genes (*MaADH1*, *MaADH2*, *MaADH7*, *MaADH6*, and *MaADH13*) had lower expression levels relative to the control group. MaADH5, *MaADH19*, and *MaADH20* maintained low relative expression under abiotic stresses, whereas *MaADH13* and *MaADH17* remained expressed at low levels under phytohormone treatments. The above results suggest that different members of this family may exhibit specific expression patterns under different stress conditions.

To validate the transcriptional responses of *MaADH* genes under flooding stress, 11 candidate genes (*MaADH1*, *MaADH3*, *MaADH4*, *MaADH5*, *MaADH6*, *MaADH7*, *MaADH11*, *MaADH13*, *MaADH14*, *MaADH17*, and *MaADH20*) showing more than a two-fold change in expression were selected for qRT-PCR analysis (Figure 10). The qRT-PCR results showed that nearly all selected *MaADH* genes were induced in roots and stems after flooding. The expression levels of most *MaADH* genes peaked at 1 day after flooding treatment, with *MaADH17* exhibiting the strongest relative expression response under flooding treatment. Next, expression levels sharply decreased, and most genes showed minimal or undetectable expression at 7 days after treatment. Compared with the strong induction observed in roots and stems, all analyzed *MaADH* genes showed a continuous decline in relative expression in leaves throughout the flooding treatment period. This contrasting tissue-specific expression pattern suggests that *MaADH* genes may primarily function in root and stem tissues during early responses to flooding-induced hypoxia.

Correlation analysis was performed to explore the relationships among expression profiles of the 11 *MaADH* genes (Figure 11). Pearson correlation coefficients were calculated in Origin 2024 software using transcriptomic FPKM values from three biological replicates. Statistically significant correlation coefficients ranged from −0.95 to 0.97, showing diverse correlated expression trends among these gene family members. A strong positive correlation was observed between *MaADH5* and *MaADH20*. However, with the exception of *MaADH14* and *MaADH5*, negative correlations were observed between *MaADH20* and most of the other tested genes. Among these correlations, the negative correlation between *MaADH20* and *MaADH17* was the strongest.

## 4. Discussion

Rapid advances in genome sequencing technologies, together with continuous improvements in bioinformatic analysis, have identified and characterized a large number of *ADH* gene families across diverse plant species. To date, numerous *ADH* genes and ADH-like sequences have been identified and functionally investigated in multiple plant families, including Poaceae, Rosaceae, Brassicaceae, Fabaceae, and Pinaceae [30]. Genome-wide identification and characterization of *ADH* gene families have been extensively conducted in various herbaceous plant species. For example, 47 *ADH* genes have been identified in *Brassica napus* [31]. Notably, 53 members have been reported in tobacco [32], and 49 *ADH* genes have been characterized in *Artemisia annua* [33]. However, comprehensive investigations of the ADH gene family in woody plant species remain comparatively limited. For example, only 15 *ADH* genes have been identified and characterized in *T. distichum* to date [15]. Variation in the number of *ADH* family members among different plant species reflects the evolutionary diversification and species-specific expansion patterns of this gene family. However, a systematic genome-wide analysis of the (*MDR*)-*ADH* gene family in *M. alba* has not been reported previously.

The evolutionary diversification of gene families is closely linked to changes in gene structure, duplication events, and functional differentiation [33]. In this study, the 20 identified *MaADH* genes showed substantial diversity in exon–intron organization, indicating structural variation among family members. Previous studies have shown that plant *ADH* genes contain nine introns [1]. Consistent with this pattern, six *MaADH* genes, including *MaADH1*, *MaADH2*, *MaADH3*, *MaADH4*, *MaADH5*, and *MaADH17*, exhibited the conserved structural pattern. Conserved motif analysis further revealed that the *MaADH* gene family possesses both highly conserved sequence characteristics and potential functional diversification. Motif2 was identified in all MaADH proteins and contained the conserved putative dinucleotide-binding motif domain, indicating that all members retain the essential structural characteristics required for fundamental oxidation–reduction reactions [34]. The zinc-binding Zn1 domain plays a critical role in coordinating the catalytic zinc ion, which facilitates substrate activation by polarizing the alcohol hydroxyl group and promotes hydride transfer during enzymatic catalysis. In this study, motif1 containing the Zn1-binding region was present in nearly all MaADH proteins, except for two members. The absence of this conserved region in these proteins suggests that these specific members may possess altered catalytic mechanisms or functional specialization compared with canonical zinc-dependent ADH proteins.

Comparative synteny analysis of *M. alba* and six representative plant species revealed that *MaADH* genes were more conserved in dicotyledonous species than in monocotyledonous species. This pattern supports a closer evolutionary relationship between mulberry and other dicot lineages. *MaADH3*, *MaADH4*, *MaADH9*, and *MaADH17* showed conserved syntenic relationships only with dicot species, whereas *MaADH14* maintained conserved synteny across all six analyzed species. These observations suggest that these genes may represent relatively conserved ancestral members of the *MaADH* family and may have retained essential biological functions throughout plant evolution.

*ADH* is widely recognized as a key enzyme in plant anaerobic fermentation, catalyzing the conversion of acetaldehyde to ethanol and contributing to plant adaptation under oxygen-limited conditions. In addition to flooding stress, *ADH*-mediated metabolic regulation has been implicated in responses to various abiotic stresses, including drought and salinity [35]. In this study, members of the *MaADH* family exhibited distinct expression patterns under the same stress conditions, suggesting that functional differentiation and differences in transcriptional regulation may exist among members of this family. These differences may reflect variations in cis-regulatory elements within the promoter region as well as differences in regulation by upstream transcription factors, such as AdRAP2.3, which has been reported to enhance flooding tolerance by promoting expression of pyruvate decarboxylase (PDC) and *ADH* genes [36]. In the non-biotic stress treatments of this study, the number of downregulated *MaADH* genes exceeded the number of induced genes, indicating that many *MaADH* members may undergo transcriptional repression during stress responses, whereas only selected members are specifically activated. Among the analyzed genes, *MaADH14* was upregulated under all three abiotic stresses, suggesting it may serve as a promising candidate for further investigation into broad-spectrum stress adaptation in mulberry. Previous studies have reported that mulberry *ADH*-encoding genes are strongly induced by flooding and may help maintain cellular energy metabolism under flooding-induced oxygen deficiency [22]. These results provide important evidence that the *MaADH* gene responds to flooding stress at the transcriptional level and lay the foundation for further studies on its functional differentiation and regulatory mechanisms. Future research should focus on using transgenic techniques to validate the functions of significantly differentially expressed genes and to identify upstream transcriptional regulators that govern their expression patterns. These studies will help elucidate the roles of these *ADH* genes in *M. alba*.

Previous studies have shown that *ADH* genes contribute to plant tolerance to various environmental stresses, including flooding, cold, drought, and salinity [15,17,18,31]. The role of ADH-mediated fermentation pathways in plant stress resistance has been well documented across multiple species [37,38,39,40]. For example, *T. distichum* shows increased ADH expression levels under flooding [15], whereas pear plants activate ADH-associated responses during hypoxic stress [41]. In this study, qRT-PCR validation showed that 11 selected *MaADH* genes were significantly induced in roots and stems under flooding stress. Within the same gene, the relative expression levels in root samples were higher than those in stem samples. Flooding stress primarily affects roots by restricting oxygen diffusion and inducing hypoxic conditions [42]. Under submerged conditions, reduced oxygen availability triggers the activation of anaerobic metabolic pathways. This study analyzes only transcriptional changes and is unable to distinguish the independent contributions of hypoxia, oxidative stress, and osmotic stress. Consistent with these observations, previous studies in *T. distichum* showed significant induction of the root-expressed *TdADHA2* and *TdADHA3* genes under flooding [15]. In flood-tolerant tree species, ethanol produced by root fermentation may be transported via the transpiration stream to aerial tissues, where it can be converted to acetyl-CoA for further metabolic use [43]. Studies in flooded grey poplar (Populus × canescens) have similarly shown enhanced glycolytic activity and ethanolic fermentation in roots but not in leaves [44]. Transcriptomic studies of chrysanthemum plants under flooding stress showed that *ADH* transcript accumulation peaked approximately 2 h after flooding, followed by a rapid decline [45]. Similarly, in five-day-old rice seedlings subjected to short-term flooding, *OsADH1* expression peaked approximately 24 h after stress exposure [46]. The *MaADH* gene in *M. alba* also exhibited a trend of initially increasing and then decreasing. These findings provide important data supporting a deeper understanding of the function of *MaADH* in the mulberry tree. Future studies could incorporate physiological, phenotypic, and enzyme activity assays to further elucidate the biological function of this gene in adaptation to flooding. Further research is needed on upstream transcription factors and regulatory networks, and the functions of key *MaADH* genes should be validated using transgenic techniques to elucidate how these *MaADHs* respond to flooding stress. Such findings provide candidate genetic resources for future research into flooding tolerance mechanisms in *M. alba*.

## 5. Conclusions

In this study, a comprehensive genome-wide analysis was conducted to characterize the (*MDR*)-*ADH* gene family in *M. alba*, identifying *20 MaADH* genes. Subcellular localization analysis predicted that most MaADH proteins are cytoplasmic. Phylogenetic assessment grouped these genes into two distinct evolutionary clades, with an uneven chromosomal distribution comprising seven chromosomes and four tandem duplication regions. Gene architecture and conserved motif analyses revealed a high level of structural conservation among family members. Comparative synteny analysis revealed that *MaADH14* had conserved collinearity across all evaluated species. Transcriptomic profiling further showed tissue-specific and stress-responsive expression patterns. These results expand current knowledge of *ADH* gene biology in woody species and provide a valuable genomic platform for future functional investigations of *MaADH* genes in *M. alba*.

## Figures and Tables

**Figure 1:**
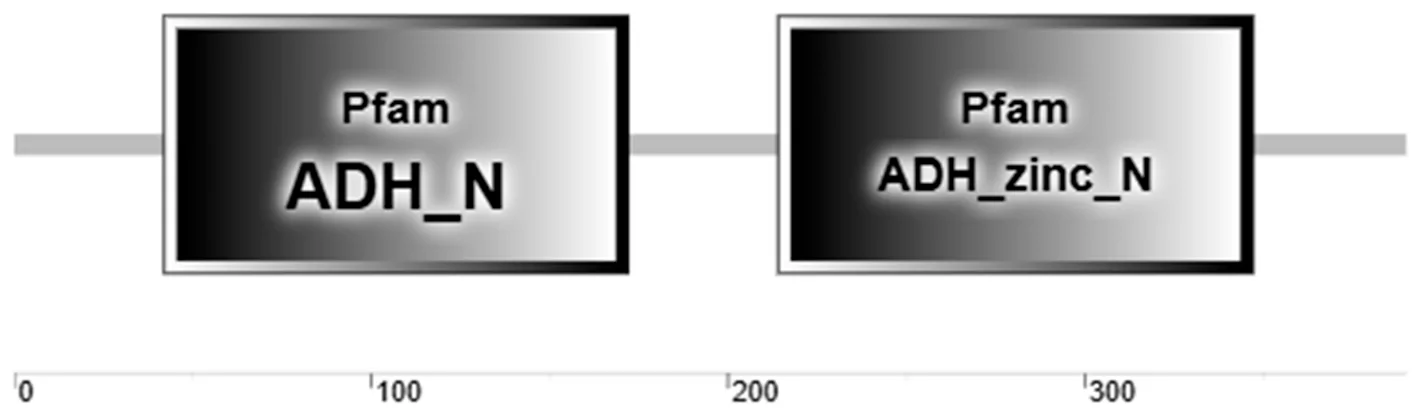
Conserved domains of the *ADH* gene. Predicting conserved domains of the ADH protein using the SMART online tool.

**Figure 2:**
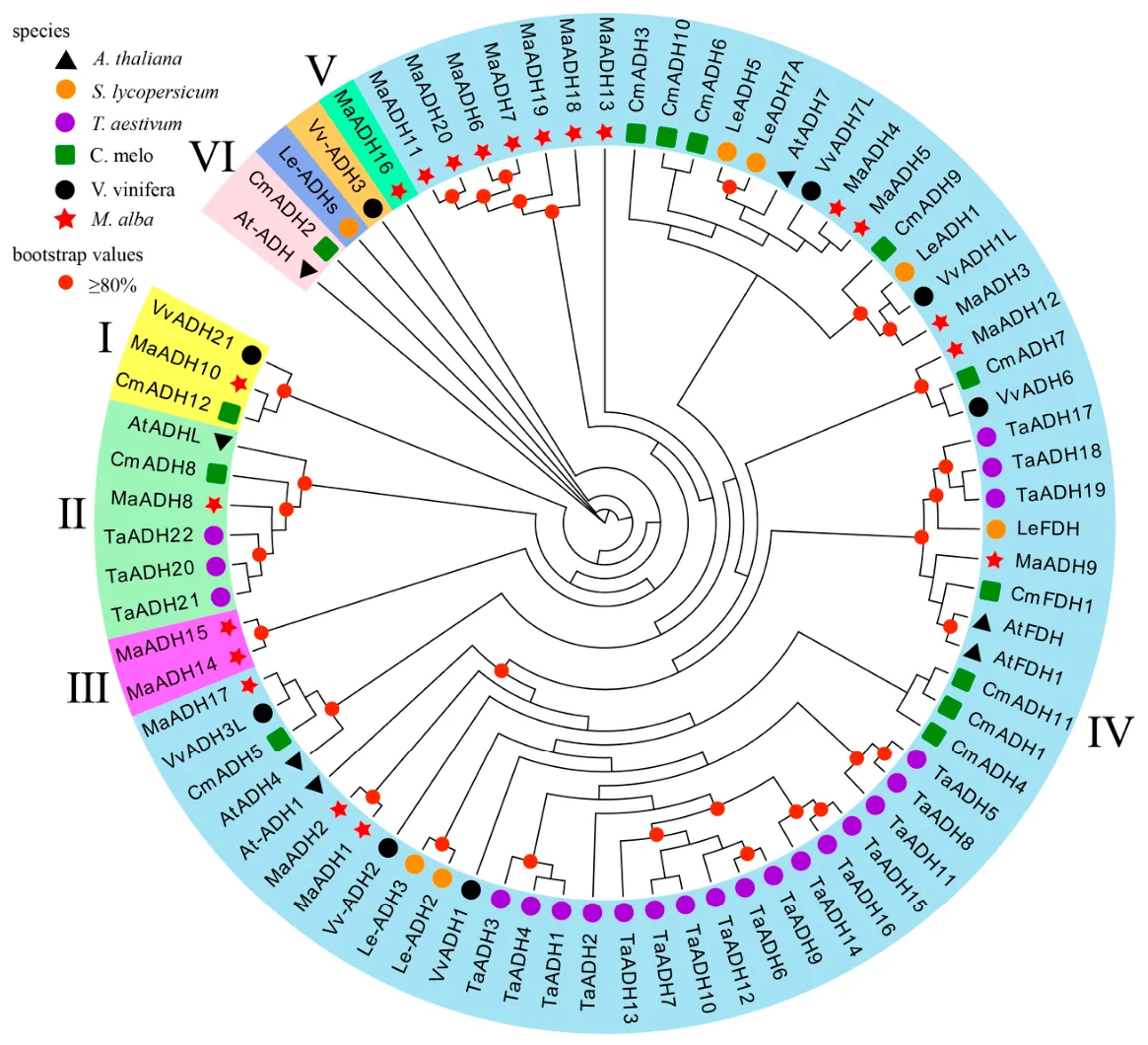
Phylogenetic relationships among MaADH proteins and ADH from five representative plant species (*A. thaliana*, *S. lycopersicum*, *T. aestivum*, *C. melo*, and *V. vinifera*). The maximum-likelihood phylogenetic tree was constructed based on a multiple sequence alignment of MaADH protein sequences using Clustal W in MEGA 7.0 (v7.0.26). Poorly aligned regions were manually trimmed. The tree was built with the LG + G4 amino acid substitution model, pairwise deletion of gaps, and 1000 bootstrap replicates. The tree is unrooted and displayed with midpoint rooting. Bootstrap values greater than 80% are shown above the branches. ADH subfamilies are indicated by distinct colors.

**Figure 3:**
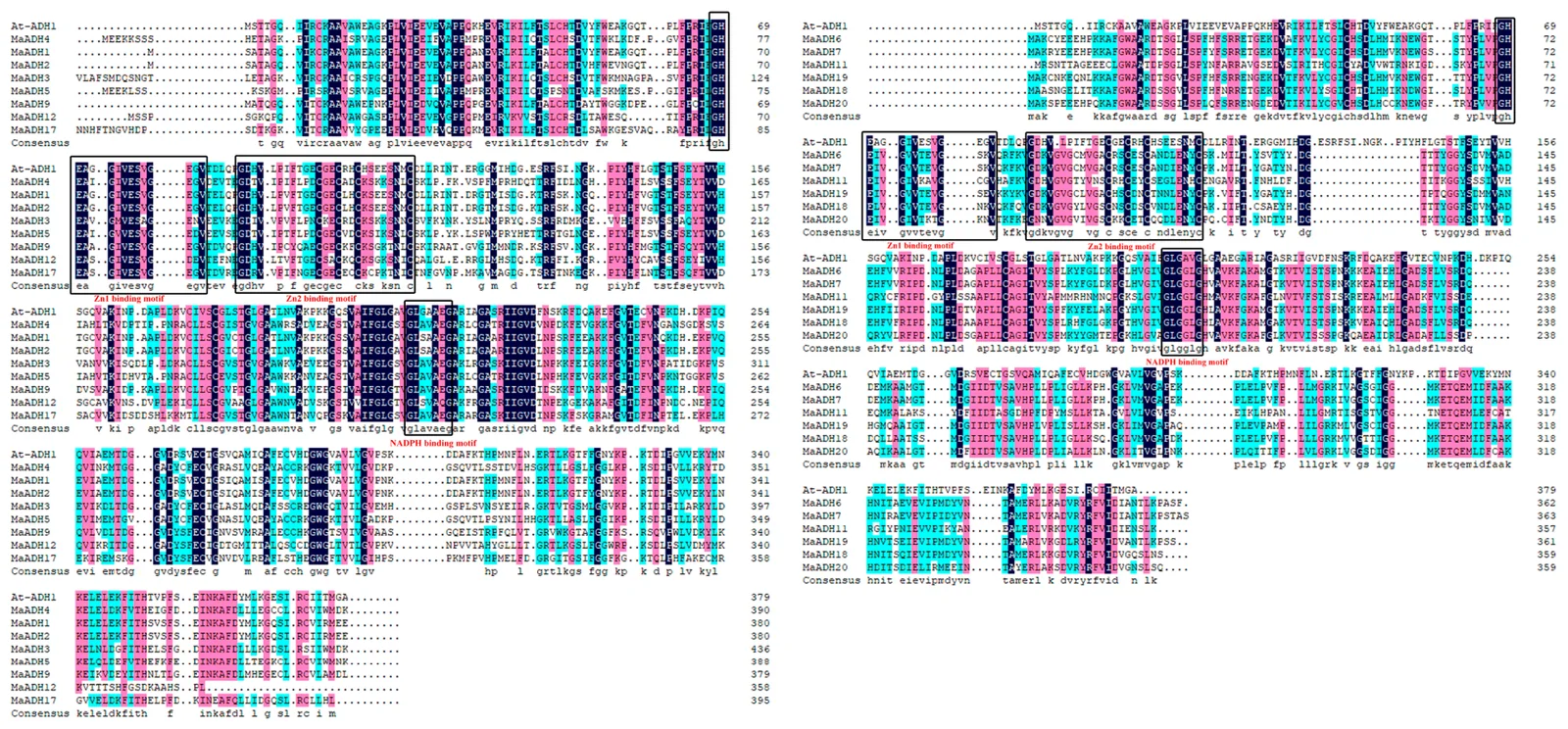
Amino acid sequence alignment of MaADH proteins. Amino acid conservation levels are indicated by background colors: black indicates 100% identity, pink indicates ≥80% identity, and blue indicates ≥60% identity. Black boxes mark the positions of the conserved Zn1, Zn2, and putative dinucleotide-binding motif domains.

**Figure 4:**
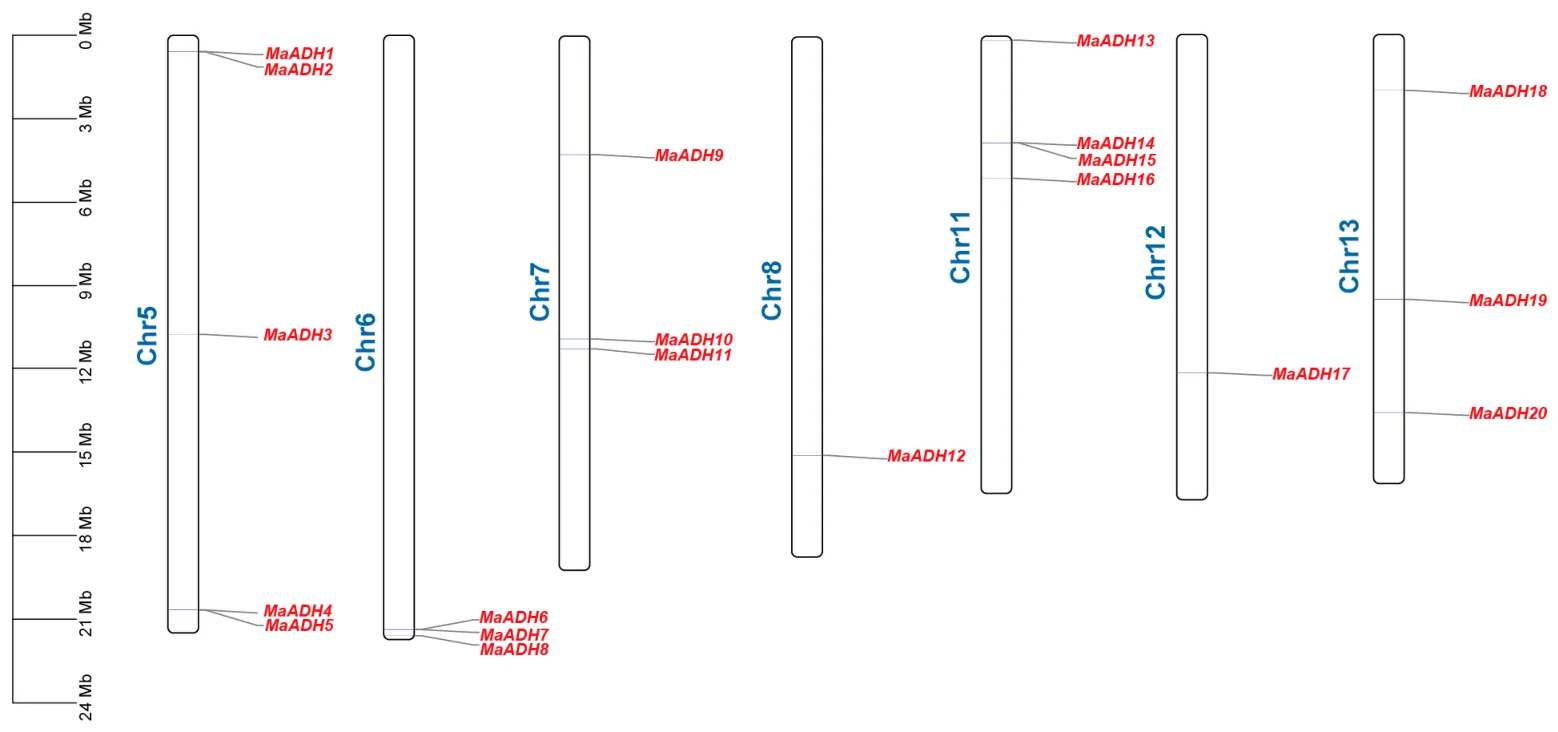
Chromosomal localization of the *MaADH* genes. Chromosome numbers are shown on the left side of each chromosome, and the scale bar indicates chromosome length in megabases (Mb).

**Figure 5:**
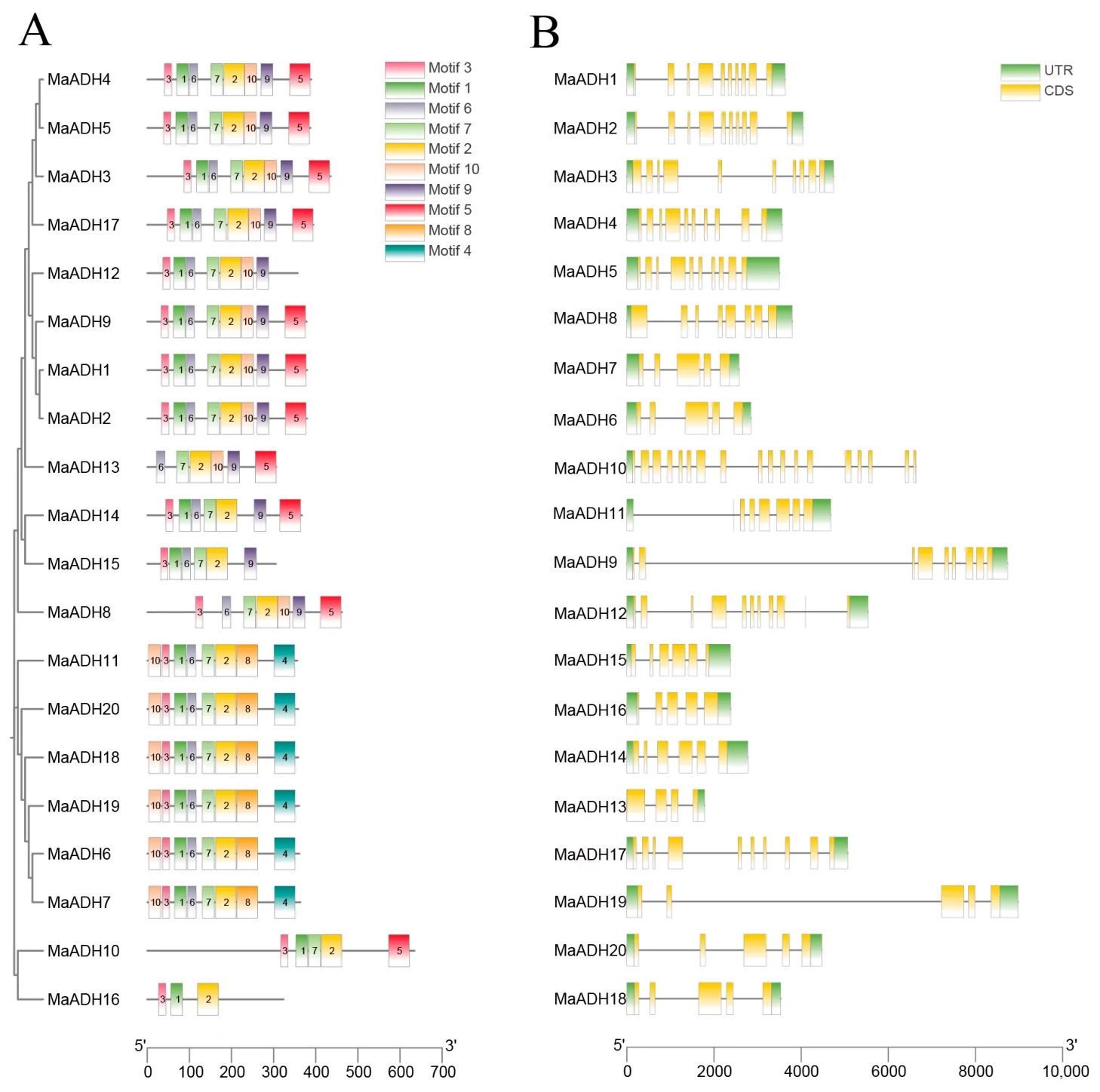
Conserved motifs and gene structures of MaADH proteins: (**A**) Conserved motif distribution patterns of MaADH protein sequences. Different colored boxes and numerical labels represent individual conserved motifs. (**B**) Exon–intron structures of *MaADH* genes. Yellow boxes represent coding sequences, green boxes represent untranslated regions (UTRs), and black lines represent intron regions.

**Figure 6:**
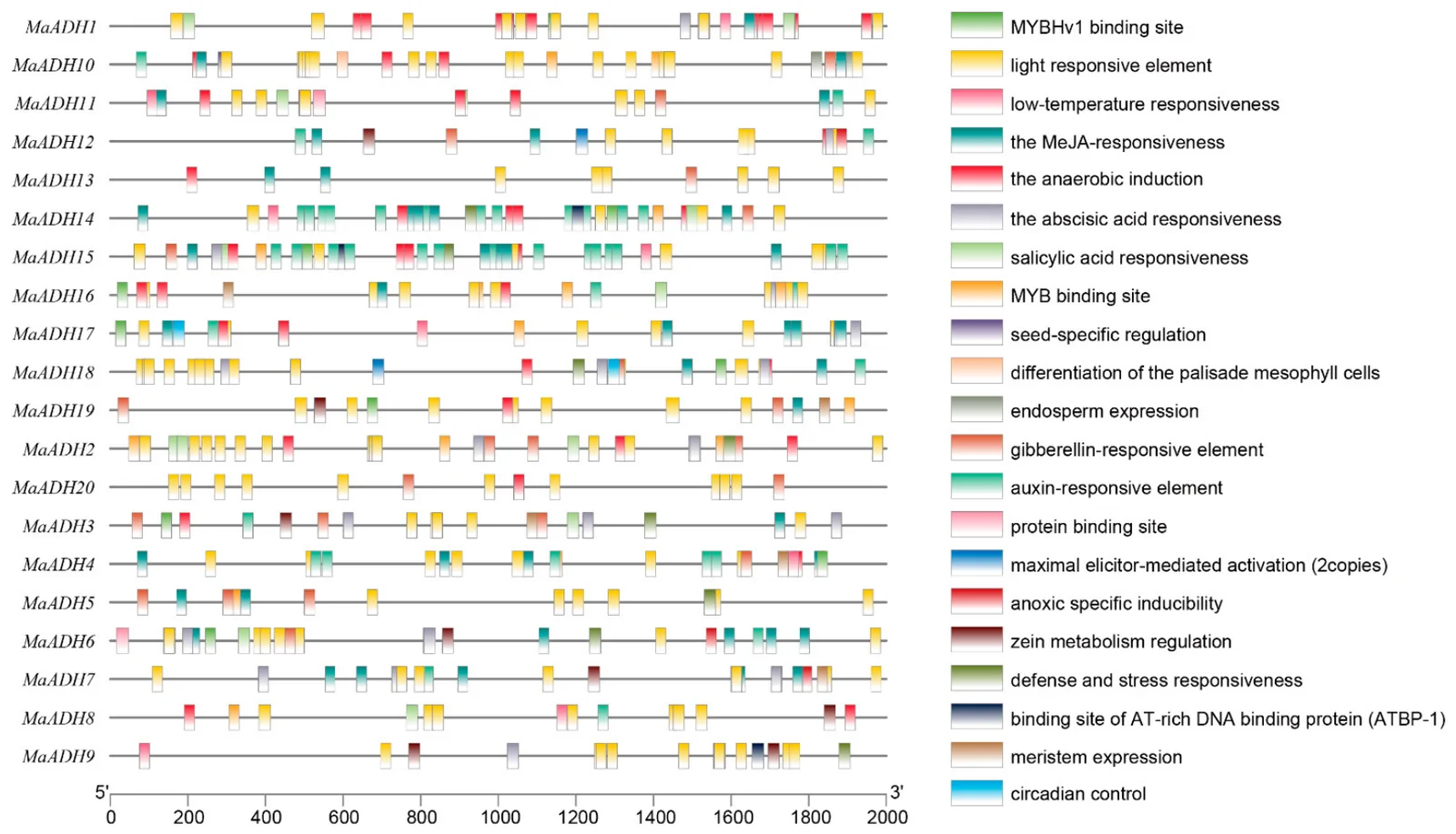
Cis-acting regulatory elements identified in the promoter regions of *MaADH* genes (2000 bp upstream of the start codon). Boxes of different colors correspond to different types of cis-acting elements.

**Figure 7:**
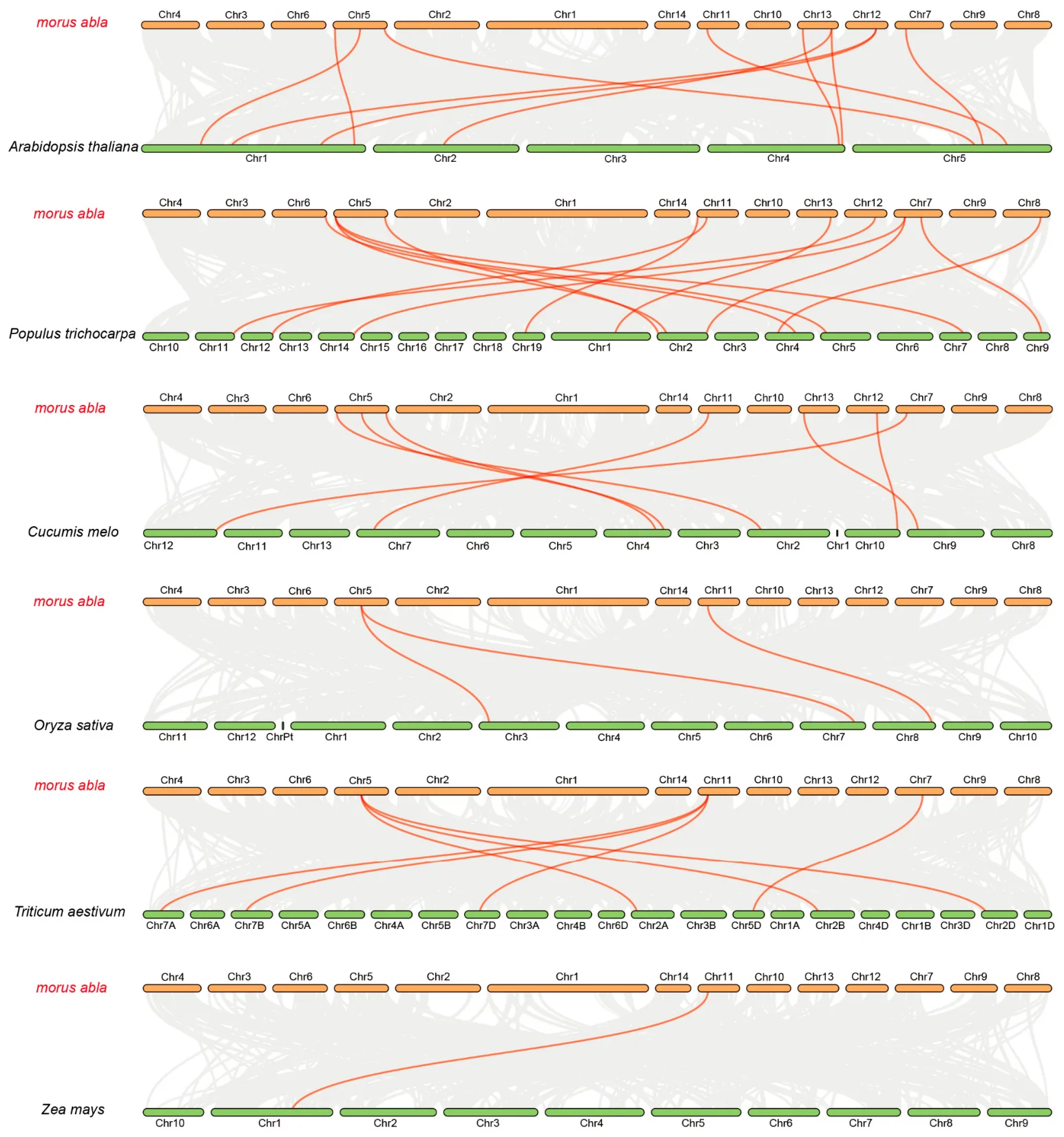
Synteny analysis of *ADH* genes between *M. alba* and six representative plant species (*A. thaliana*, *P. trichocarpa*, *C. melo*, *O. sativa*, *T. aestivum*, and *Z. mays*). Gray lines represent conserved syntenic blocks between the *M. alba* genome and the genomes of the compared species. Red lines indicate orthologous relationships between *MaADH* genes and corresponding homologous genes.

**Figure 8:**
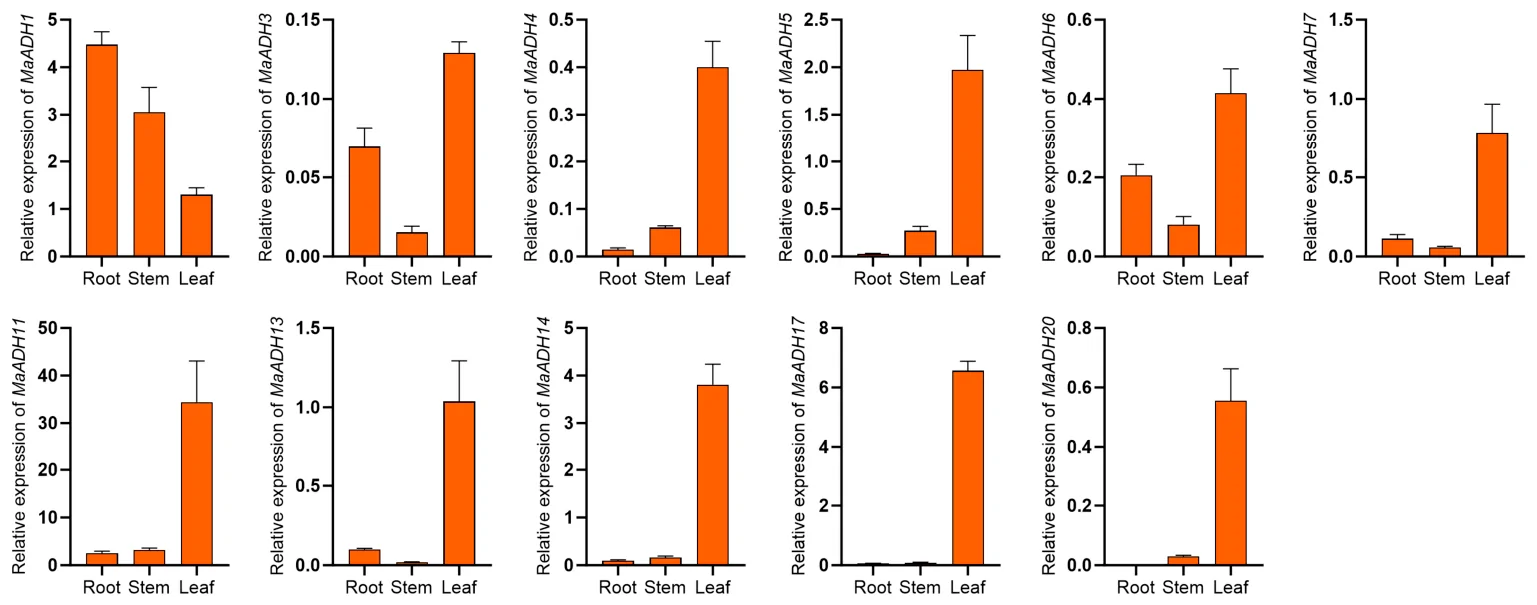
Tissue expression profiles of 11 *MaADH* genes in the roots, stems, and leaves of mulberry trees under normal conditions (0 days). Relative expression was calculated using the 2^−ΔCt^ method. Data represent mean ± SD. Three biological replicates were used for each treatment.

**Figure 9:**
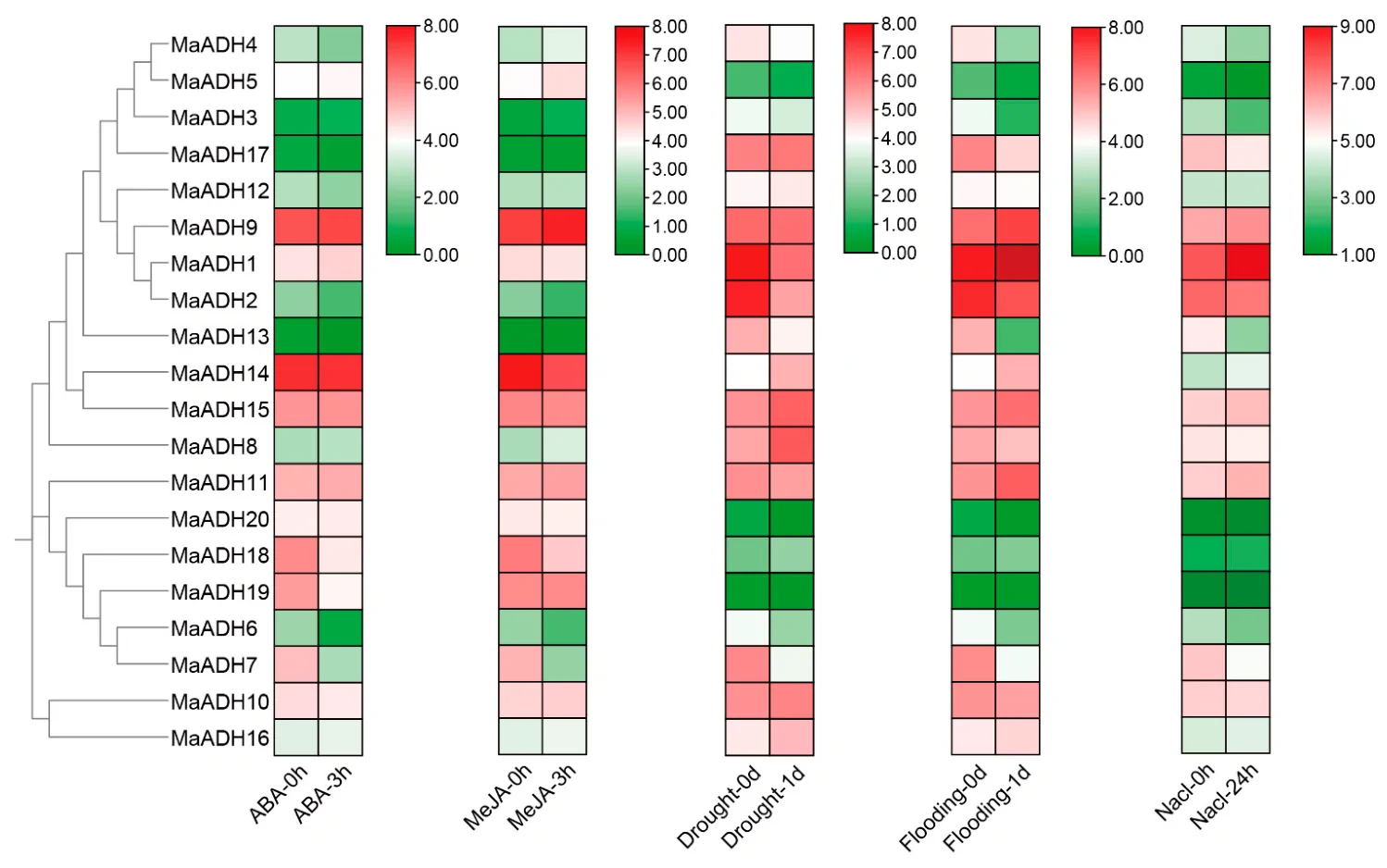
*MaADH* gene expression profile based on transcriptomic FPKM values. The color scale represents relative expression levels from low (green) to high (red).

**Figure 10:**
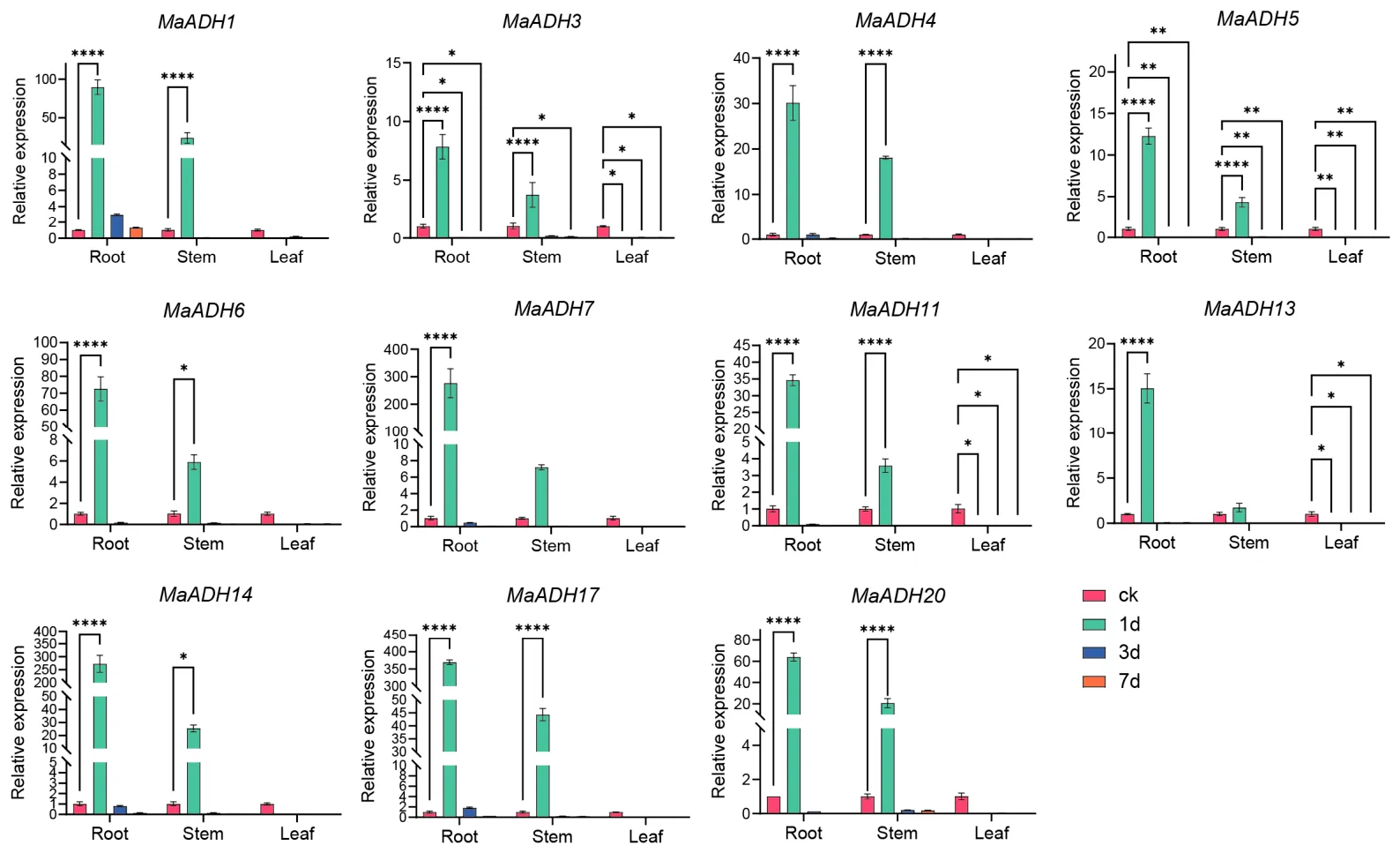
Expression patterns of *MaADH* genes under flooding stress. Using untreated tissue as a control, we analyzed gene expression in roots, stems, and leaves at 1, 3, and 7 days under flooding conditions. Total RNA was isolated from the roots, stems, and leaves of mulberry seedlings, and the expression levels of 11 *MaADH* genes were detected using qPCR and normalized against an internal control gene. Values represent mean ± standard deviation. Asterisks indicate statistically significant differences: * *p* ≤ 0.05, ** *p* ≤ 0.01, **** *p* ≤ 0.0001. Three biological replicates were used for each treatment.

**Figure 11:**
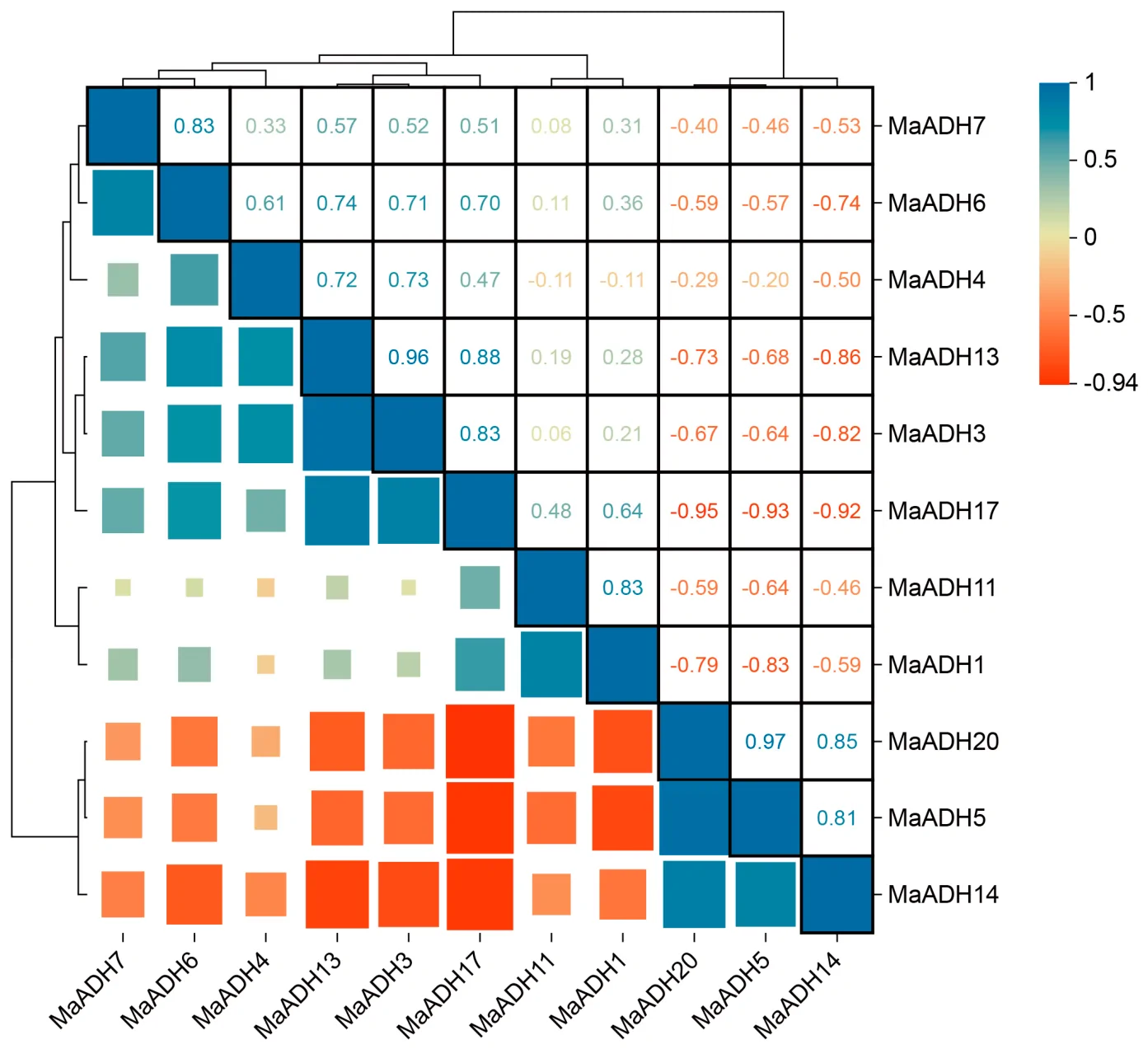
Correlation analysis of *MaADH* gene expression profiles under different stress treatments. Correlation coefficients are represented using a color gradient, with positive correlations indicated in blue and negative correlations indicated in red. The color scale ranges from −0.95 to 1.00 and represents standardized correlation coefficients for gene expression levels.

## Data Availability

The RNA-seq data are available at NCBI SRA (https://www.ncbi.nlm.nih.gov/sra, accessed on 18 September 2026) and will be made public one month after the article is published under accession SRP737661. Further inquiries can be directed to the corresponding author.

## Supplementary Materials

The following supporting information can be downloaded at https://www.mdpi.com/article/10.3390/biology15201808/s1, all supplementary figures are compiled in a single Microsoft Word file (supplementary Figures): Figure S1: Sequence logos for conserved motifs; Figure S2: Number of cis-regulatory elements; Figure S3: Heatmap of cis-regulatory elements. Table S1: Transcriptomic data for the *MaADH* genes; Table S2: qPCR Specific Primer Sequence Information; Table S3: Physicochemical properties of *MaADH* genes; Table S4: Other species *ADH* information; Table S5: Conserved Motif Analysis in MaADH proteins; Table S6: List of the identified cis-regulatory elements; Table S7: Collinearity analysis of the *ADH* genes.
