# Supplementary material for: Comprehensive Bioinformatic Analysis and Expression Profiling of the Alcohol Dehydrogenase (ADH) Gene Family in Morus alba
Source: Biology (Basel). 2026 Oct 10;15(20):1808. doi: 10.3390/biology15201808 (PMC13702005; doi:10.3390/biology15201808)
Supplement: Supplementary file 1 [file biology-15-01808-s001.zip › Supplementary Figures.pdf]

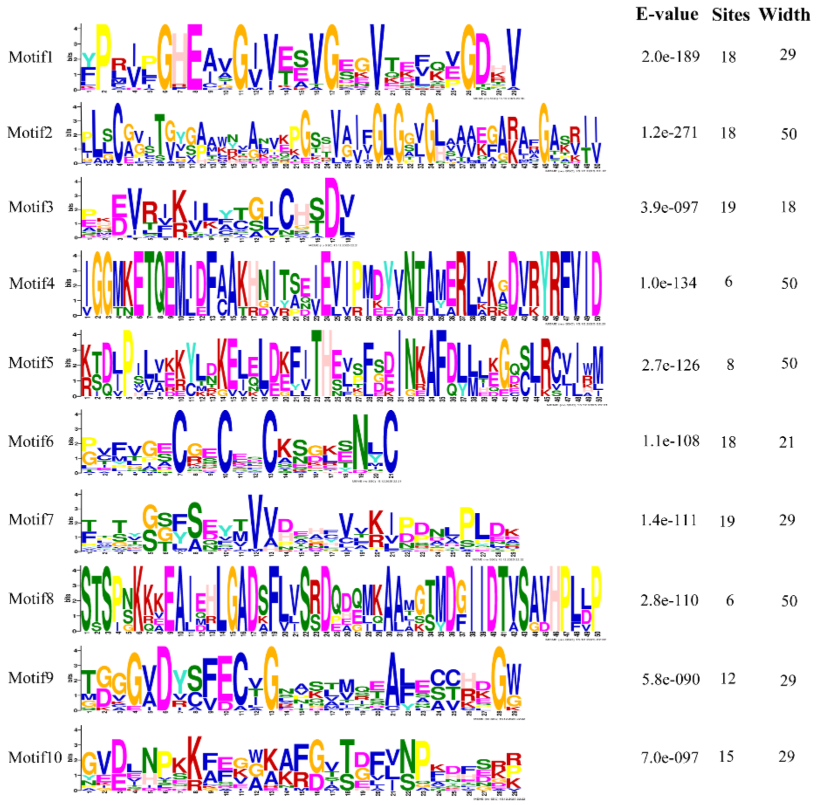


**Figure S1.** Sequence logo of conserved motifs in the MaADH protein.

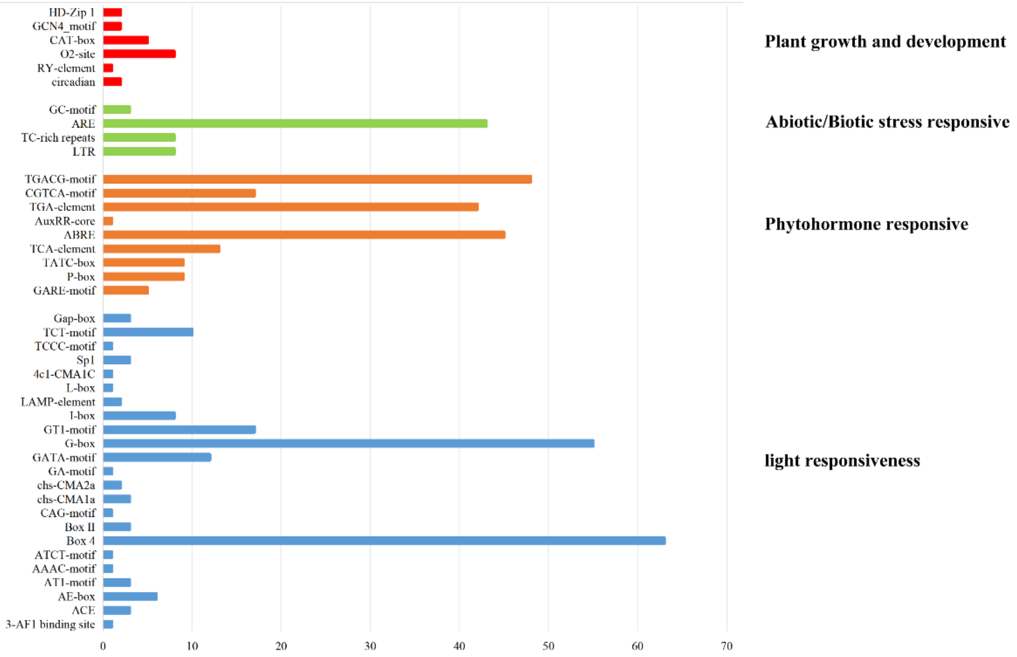


**Figure S2.** Number of cis-regulatory elements detected in the promoter regions of *MaADH* genes. The names of the cis-acting elements are shown on the left of the panel, with their

corresponding functional annotations listed on the right.

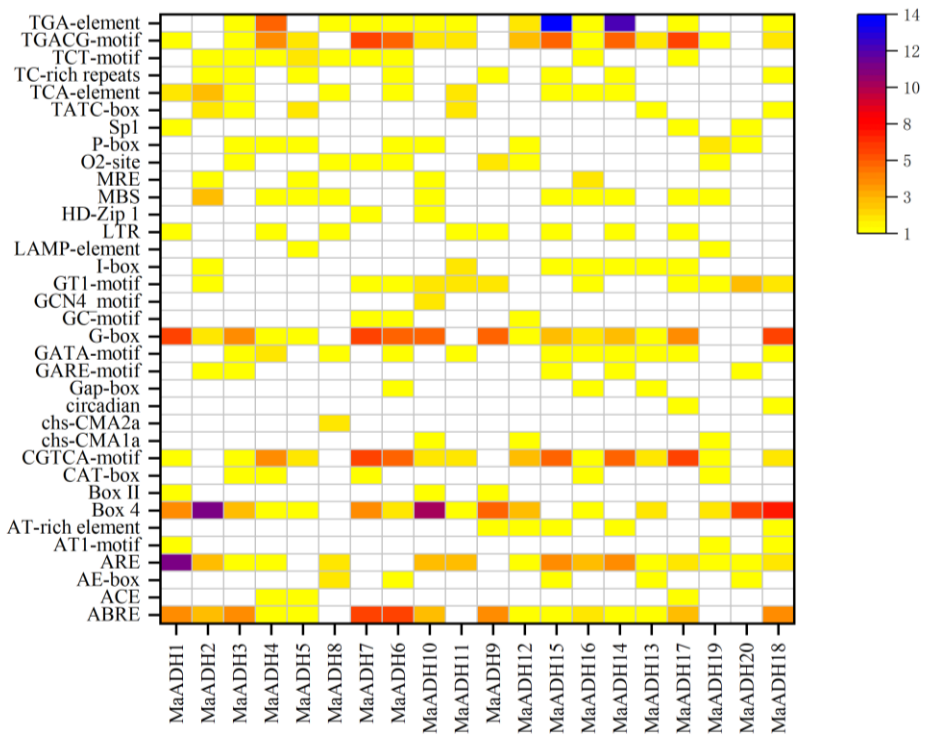


**Figure S3.** Cis-regulatory element analysis of the promoter regions of *MaADH* genes.
